# Cisplatin as neoadjuvant chemotherapy in triple negative breast cancer: Exciting early results

**DOI:** 10.4103/0971-5851.73588

**Published:** 2010

**Authors:** Arun Kumar Goel, Malay Nandy, Gopal Sharma

**Affiliations:** *Department of Surgical Oncology, Galaxy Cancer Institute, Pushpanjali Crosslay Hospital, W-3, Sector 1, Vaishali, Gaziabad, Uttar Pradesh, India. E-mail: akg.surgonc@galaxycancerinstitute.com*; 1 *Department of Medical Oncology, Galaxy Cancer Institute, Pushpanjali Crosslay Hospital, W-3, Sector 1, Vaishali, Gaziabad, Uttar Pradesh, India*

The approach to breast cancer management has undergone a dramatic shift. As near as a decade ago, clinical TNM stage was the main determinant of the broad treatment plan. Pathological information available after surgery, including hormone receptor status, was used to complement TNM stage group for making treatment decisions. In contrast, molecular markers are now playing a progressively increasing role in planning treatment. This is one of the main reasons that a core needle biopsy for histological examination and assessment of estrogen receptor, progesterone receptor and ERBB2/HER 2 is being used widely in place of fine needle aspiration cytology as a pre-treatment investigation.

Breast cancers are now categorized into many subtypes: normal breast like, luminal A, luminal B, ERBB2 + and basal like. The definition of these subtypes is based on gene expression profiling and has a strong correlation with the prognosis of disease.[1,2] The biological basis of the different subtypes has been considered. Gene expression patterns that are similar to the malignant subtypes have been found in different areas of the breast duct/lobular system.[3,4] Whether the different expression patterns are related to differences in cell of origin or to different pathways of carcinogenesis is not yet clear.

In addition to a guide to prognosis, the role of these subtypes in predicting responses to different types of treatment is being keenly studied. Tumors that are expressing hormone receptors belong to the luminal groups. Luminal A type tumors have high levels of estrogen receptor expression. Tumors expressing hormone receptors at high levels are generally slow growing, respond well to hormonal interventions (tamoxifen, aromatase inhibitors, Luteinizing hormone releasing hormone (LHRH) analogues) and show a lesser response to chemotherapy.[5] The other three categories (normal breast like, basal like and ERBB2 +) are poor or lacking in estrogen receptor expression. Of these, tumors that overexpress ERBB2 and do not express hormone receptors generally are faster growing, do not respond to hormonal intervention, and respond to chemotherapy drugs (with a higher sensitivity to anthracyclines). Addition of trastuzumab to standard chemotherapy leads to a significant benefit for this group of patients.[6,7]

The subgroup of Triple Negative breast Cancers (TNBC) has attracted a lot of attention in recent years. Firstly, this group of tumors derives no benefit from either hormonal interventions or with trastuzumab, two targeted approaches currently available in breast cancer management. Further, these tumors are high grade with a higher early recurrence rate. The subgroup of basal like cancers on gene profiling studies is usually triple negative by immunohistochemistry. The two terms (TNBC and basal like) are however not synonymous.[8,9] Another interesting observation is that patients with familial breast cancer related to BRCA 1 gene abnormalities have a basal like gene profile.[10,11] Because of this finding, it is suspected that basal like tumor without germline mutations in the BRCA 1 gene may have somatic mutations or inactivation of the BRCA 1 pathway. Based on this background information, it is being considered that BRCA 1 related familial breast cancers, basal like breast cancers on genomic profiling and triple negative breast cancers may have significant commonalities and similar investigative approaches may be productive in all of them.

Management of triple negative breast cancers relies solely on chemotherapy.[12] There is a state of uncertainty and confusion about the management of these cancers. It is commonly thought that TNBC are aggressive in behavior and have a poor outcome compared to other type of breast cancers. However, there is some evidence to suggest that the response of TNBC to first-line chemotherapy is good and may be better than other groups, i.e. HER2 overexpressing or ER/PR expressing breast cancers. Also, the TNBC patients who have a complete response to neoadjuvant chemotherapy seem to have very good long-term survival.[13] At the same time, it is still not clear as to what is the best chemotherapy regimen for TNBC patients. Many trials are underway.[3] Currently, the same chemotherapy regimens are used for these patients as for other breast cancer subtypes. Further, TNBC patients do not have very effective second- and third-line chemotherapy agents.

With this background, lot of research work is currently underway to identify newer targets for systemic therapy of TNBC. Antiangiogenic agents are under investigation in breast cancer management, including management of TNBC.[14] An interesting line of investigation comes from the similarity with BRCA 1 related cancers. BRCA 1 gene product is involved in DNA repair pathways, specifically homologous recombination of double strand breaks. It is felt that basal like cancers are not hereditary and by extension of the same logic, triple negative breast cancers may be having defects in the DNA damage response pathways. It is thus postulated that some DNA damaging agents may be more effective in this type of tumors. The platinum agents are classically known to induce DNA double strand breaks. Cisplatin and other platinum compounds are thus likely to be more effective in this group of tumors. Very early clinical data are becoming available to explore this hypothesis. One small study from Poland has reported cisplatin as a single agent neoadjuvant therapy in breast cancers with the background of germline BRCA 1 mutations.[15] Pathological complete response rates of more than 80% have been observed with four cycles of single agent cisplatin. In fact, two of the patients achieved pathological complete responses with only two cycles of cisplatin. This small study suggests that BRCA 1 related breast cancers may be exquisitely sensitive to cisplatin and probably other platinum agents. A recent study has been reported from USA in the Journal of Clinical Oncology. In this study, cisplatin was used as a single agent neoadjuvant therapy in triple negative breast cancers.[16] Two patients in this cohort were BRCA 1 related and both the patients had pathological complete responses with four cycles of cisplatin. Though these data are highly promising and exciting, it still cannot form the basis of treatment recommendations. Larger phase III studies are necessary to document the benefit of platinum agents and define a clear-cut management strategy.

The group of triple negative breast cancers that are unrelated to BRCA 1 germline mutations also needs to be assessed. The study published in Journal of Clinical Oncology has addressed this group of patients.[16] In this study, 28 patients with triple negative breast cancer were treated with single agent cisplatin as neoadjuvant chemotherapy given for four cycles. This was followed by surgery and adjuvant therapy. Adjuvant chemotherapy was based on the standard treatment guidelines. Out of the 28 patients, 2 had BRCA 1 germline mutations. Overall, six patients had complete pathological response (21%). Both the patients with BRCA 1 mutations had complete pathological response. Out of the remaining 26, four had complete response and eight had a good pathological response. DNA typing was done for 24 of 28 tumors and all belonged to the basal like group.

Based on these observations, it appears that cisplatin is a highly active single agent in triple negative breast cancers.[15,16] In BRCA 1 germline mutation carriers, the sensitivity seems to be very high with complete pathological responses seen in majority of patients and even two cycles of cisplatin leading to complete pathological responses in two patients. In patients without BRCA 1 germline mutations, the complete response rates are lower (4/26; 15% approximately). However, it is still important to highlight that for neoadjuvant chemotherapy based on a single drug, even a 15% complete pathological response is good.

The above observations only mark the beginning of a new era in the management of triple negative/basal/hereditary breast cancer. These results need to be validated in a larger cohort of patients. Attempts to identify predictors of response were made in the above-mentioned study but no significant associations could be identified.[16] This process would need to be repeated with a wider array of possible predictors.

Another important development has been identification of poly ADP ribose polymerase (PARP) inhibitors as another group of drugs with significant activity in this group of patients.[17] PARP is an enzyme that plays an important role in DNA repair pathways. The activity is important for single strand repair pathway. Patients with BRCA 1 related tumors already have defects in double strand break repairs. Inhibition of PARP and impairment of single strand repair pathway increases the demand on the double strand break repair pathway. With pre-existing defects in the pathway, DNA repair caused by chemotherapy drugs does not get repaired, increasing the cell kill.

Future studies would have to identify combination of targeted therapy with conventional chemotherapy drugs, e.g., cisplatin and achieve higher complete response rates and thus improve long-term survival in this group of patients. A study has been reported using a combination of cisplatin and bevacizumab as neoadjuvant therapy.[18] Approximately 37% patients had a complete or near complete pathological response (19/45). Six patients in the study did not complete the planned neoadjuvant treatment due to toxicity.

A cautionary note is essential because these results are early and such treatment approaches are not recommended outside trial settings. However, these results are a pointer to the direction for breast cancer management in the future. It is expected that in a not so distant future, the best approach for treatment of a given patient will be based on a combination of information that consists of clinical and radiological stage of tumor, molecular profile of the cancer and patient factors and patients’ choice. A better understanding of the molecular basis of breast cancer and identification of additional molecular targets would lead to the optimal utilization of targeted therapy in combination with conventional cytotoxic drugs. Further, availability of high throughput molecular profiling technologies is expected to make it feasible and affordable for individual patients, allowing for individualized treatment prescriptions.
